# Methyl 3-[(chloro­meth­oxy)carbon­yloxy]-7-hy­droxy­cholan-24-oate

**DOI:** 10.1107/S160053681301725X

**Published:** 2013-06-29

**Authors:** Jia-Liang Zhong, Wen-Xia Sun, Fu-Li Zhang, Li-Hong Liu, He Liu

**Affiliations:** a Shanghai Institute of Pharmaceutical Industry, Shanghai 200040, People’s Republic of China; b Beijing Chao-Yang Hospital Affiliated with Beijing Capital Medical University, Beijing 100020, People’s Republic of China

## Abstract

The title compound, C_27_H_43_ClO_6_, is a derivative of urso­deoxy­cholic acid, in which the OH group at the 3-position is substituted by a chloro­meth­oxy­carbon­yloxy substituent and the carb­oxy­lic acid group at the 24-position is methyl­ated. The *A* and *B* rings are *cis*-fused, while all other rings are *trans*-fused. In the crystal, two adjacent mol­ecules located along the *b*-axis direction are inter­locked head-to-tail due to weak C—H⋯O hydrogen bonds. Therefore each mol­ecule is linked to four neighbouring mol­ecules by four C—H⋯O hydrogen bonds, with the OH group at the 7-position and the carbonyl O atom of the ester group acting as the acceptor sites.

## Related literature

For the synthesis of the title compound, see: von Geldern *et al.* (2004 ▶); For similar structures, see: Kannan *et al.* (2001 ▶); Lindley & Carey *et al.* (1987 ▶).
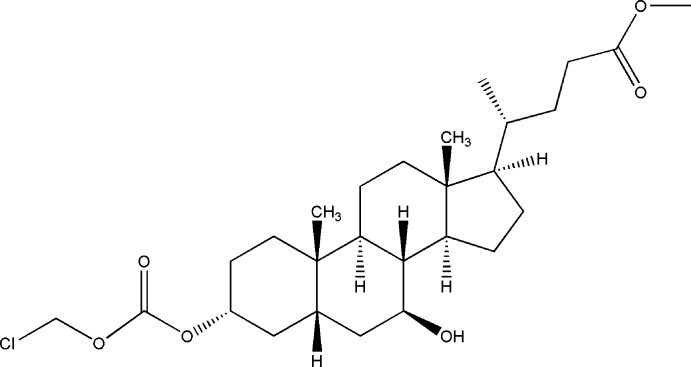


## Experimental

### 

#### Crystal data

C_27_H_43_ClO_6_
*M* *_r_* = 499.06
Orthorhombic, 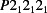

*a* = 7.8896 (13) Å
*b* = 10.9493 (17) Å
*c* = 30.683 (5) Å
*V* = 2650.6 (7) Å^3^
*Z* = 4
Mo *K*α radiation
μ = 0.18 mm^−1^
*T* = 294 K
0.24 × 0.20 × 0.12 mm

#### Data collection

Bruker APEXII CCD diffractometer
15292 measured reflections
5411 independent reflections
3191 reflections with *I* > 2σ(*I*)
*R*_int_ = 0.054

#### Refinement

*R*[*F* ^2^ > 2σ(*F* ^2^)] = 0.050
*wR*(*F* ^2^) = 0.118
*S* = 0.94
5411 reflections
311 parameters
H-atom parameters constrained
Δρ_max_ = 0.15 e Å^−3^
Δρ_min_ = −0.19 e Å^−3^
Absolute structure: Flack (1983 ▶), 2309 Friedel pairs
Flack parameter: −0.10 (9)

### 

Data collection: *APEX2* (Bruker, 2009 ▶); cell refinement: *SAINT* (Bruker, 2009 ▶); data reduction: *SAINT*; program(s) used to solve structure: *SHELXS97* (Sheldrick, 2008 ▶); program(s) used to refine structure: *SHELXL97* (Sheldrick, 2008 ▶); molecular graphics: *SHELXTL* (Sheldrick, 2008 ▶); software used to prepare material for publication: *publCIF* (Westrip, 2010) ▶.

## Supplementary Material

Crystal structure: contains datablock(s) . DOI: 10.1107/S160053681301725X/im2434sup1.cif

Structure factors: contains datablock(s) I. DOI: 10.1107/S160053681301725X/im2434Isup2.hkl

Additional supplementary materials:  crystallographic information; 3D view; checkCIF report

## Figures and Tables

**Table 1:** Hydrogen-bond geometry (Å, °)

*D*—H⋯*A*	*D*—H	H⋯*A*	*D*⋯*A*	*D*—H⋯*A*
C11—H11*B*⋯O2^i^	0.97	2.49	3.339 (4)	146
C27—H27*B*⋯O5^ii^	0.97	2.40	3.270 (4)	149

Symmetry codes: (i) 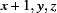; (ii) 
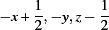
.

**Fig. 1.**
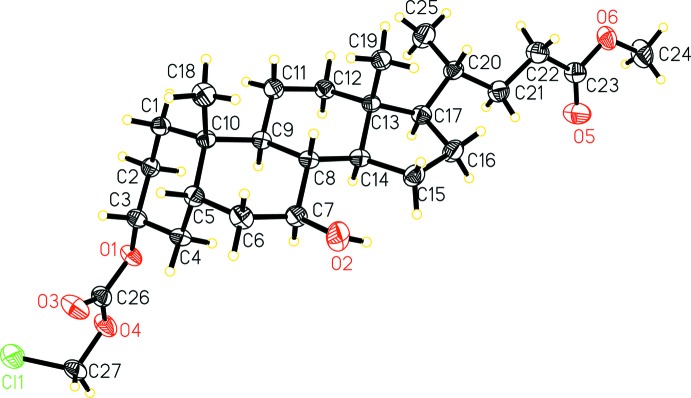
Molecular structure of (I) showing the atom-labelling scheme. Displacement ellipsoids are drawn at the 30% probability level.

**Fig. 2.**
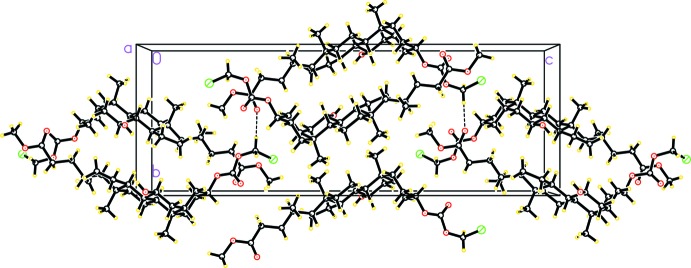
The packing of the title compound, viewed down the a axis. Dashed lines indicate the C—H···O hydrogen bonds.

## supplementary crystallographic information

### Comment

The title compound, (I) (Fig. 1), methyl 3-((chloromethoxy)carbonyloxy-7- hydroxycholan-24-oate was synthesized according to a general literature procedure (von Geldern *et al.*, 2004).

Bile acid is a widely studied and used pharmaceutical molecule (Kannan *et al.*, 2001). It can be used to treat jaundice, gallstones (Lindley & Carey, 1987) *etc*. Ursodeoxycholic acid (UDCA) and chenodeoxycholic acid (CDCA) are two most famous analogues of bile acid. Here we report the crystal structure of a UDCA derivative.

In the crystal structure, rings A and B are *cis* fused while rings B/C/D are *trans* fused. The dihedral angles of A/B, B/C, C/D are 62.89 (8)°, 2.70 (14)° and 5.05 (17)°, respectively. So the skeleton of the title compound exhibits a V shape with the 3-α and 17-β side chains stretched as the arms. In the crystal packing (Fig. 2), two adjacent molecules located along the *b* axis are interlocked head to tail due to weak hydrogen bondings (C11—H11B···O2 and C27—H27B···O5, Table 1). Therefore each molecule of the title compound is linked to four neighboring molecules by four C—H···O hydrogen bonds.

### Experimental

Chloromethyl-chloroformate was slowly added to a solution of methyl 3,7- dihydroxycholan-24-oate and anhydrous pyridine in anhydrous CH_2_Cl_2_ under nitrogen at 273 K. The resulting mixture was allowed to warm to room temperature, was then stirred for 7 h and then extracted with CH_2_Cl_2_. The organic extract was dried over Na_2_SO_4_, filtered and evaporated under reduced pressure. The residue was washed with diethyl ether to afford the title compound as a white solid (>95% yield). No further purification was necessary. Crystals appropriate for X-ray diffraction data collection were obtained by slow evaporation of a saturated DMF/H_2_O solution at room temperature.

### Refinement

The H atom of the OH-group in 7-position was located from the difference Fourier map and restraind to ride on its parent O atom. All other H atoms were placed in geometically idealized positions and constrained to ride on their parent atoms with C—H distances of 0.93 Å (0.96 for methyl group) and *U*_iso_(H) = 1.2 (1.5 for CH_3_)*U*_eq_(C) for CH.

### Crystal data

**Table:**

C_27_H_43_ClO_6_	*F*(000) = 1080
*M**_r_* = 499.06	*D*_x_ = 1.251 Mg m^−^^3^
Orthorhombic, *P*2_1_2_1_2_1_	Mo *K*α radiation, λ = 0.71073 Å
Hall symbol: P 2ac 2ab	Cell parameters from 2869 reflections
*a* = 7.8896 (13) Å	θ = 2.3–20.2°
*b* = 10.9493 (17) Å	µ = 0.18 mm^−^^1^
*c* = 30.683 (5) Å	*T* = 294 K
*V* = 2650.6 (7) Å^3^	Block, colorless
*Z* = 4	0.24 × 0.20 × 0.12 mm

### Data collection

**Table:**

Bruker APEXII CCD diffractometer	3191 reflections with *I* > 2σ(*I*)
Radiation source: fine-focus sealed tube	*R*_int_ = 0.054
Graphite monochromator	θ_max_ = 26.4°, θ_min_ = 2.0°
φ and ω scans	*h* = −9→9
15292 measured reflections	*k* = −13→13
5411 independent reflections	*l* = −38→24

### Refinement

**Table:**

Refinement on *F*^2^	Secondary atom site location: difference Fourier map
Least-squares matrix: full	Hydrogen site location: inferred from neighbouring sites
*R*[*F*^2^ > 2σ(*F*^2^)] = 0.050	H-atom parameters constrained
*wR*(*F*^2^) = 0.118	*w* = 1/[σ^2^(*F*_o_^2^) + (0.0543*P*)^2^ + 0.1843*P*] where *P* = (*F*_o_^2^ + 2*F*_c_^2^)/3
*S* = 0.94	(Δ/σ)_max_ = 0.006
5411 reflections	Δρ_max_ = 0.15 e Å^−^^3^
311 parameters	Δρ_min_ = −0.19 e Å^−^^3^
0 restraints	Absolute structure: Flack (1983), 2309 Friedel pairs
Primary atom site location: structure-invariant direct methods	Flack parameter: −0.10 (9)

### Special details

**Table:**

Geometry. All e.s.d.'s (except the e.s.d. in the dihedral angle between two l.s. planes) are estimated using the full covariance matrix. The cell e.s.d.'s are taken into account individually in the estimation of e.s.d.'s in distances, angles and torsion angles; correlations between e.s.d.'s in cell parameters are only used when they are defined by crystal symmetry. An approximate (isotropic) treatment of cell e.s.d.'s is used for estimating e.s.d.'s involving l.s. planes.
Refinement. Refinement of *F*^2^ against ALL reflections. The weighted *R*-factor *wR* and goodness of fit *S* are based on *F*^2^, conventional *R*-factors *R* are based on *F*, with *F* set to zero for negative *F*^2^. The threshold expression of *F*^2^ > σ(*F*^2^) is used only for calculating *R*-factors(gt) *etc*. and is not relevant to the choice of reflections for refinement. *R*-factors based on *F*^2^ are statistically about twice as large as those based on *F*, and *R*- factors based on ALL data will be even larger.

### Fractional atomic coordinates and isotropic or equivalent isotropic displacement parameters (Å^2^)

**Table:**

	*x*	*y*	*z*	*U*_iso_*/*U*_eq_	
Cl1	0.32107 (15)	0.26524 (10)	0.18096 (3)	0.0899 (4)	
O1	0.2665 (3)	0.37444 (17)	0.31505 (6)	0.0499 (5)	
O2	−0.3225 (2)	0.4663 (2)	0.46546 (7)	0.0736 (7)	
H2	−0.3270	0.4100	0.4831	0.110*	
O3	0.1100 (3)	0.4228 (2)	0.25576 (7)	0.0717 (7)	
O4	0.2450 (3)	0.24219 (18)	0.26367 (6)	0.0657 (7)	
O5	0.1643 (4)	0.0724 (2)	0.72053 (8)	0.0823 (8)	
O6	0.2415 (3)	0.18771 (19)	0.77669 (7)	0.0623 (6)	
C1	0.3092 (4)	0.6337 (2)	0.39347 (9)	0.0435 (7)	
H1A	0.3016	0.7011	0.3730	0.052*	
H1B	0.3992	0.6527	0.4139	0.052*	
C2	0.3587 (3)	0.5194 (2)	0.36840 (9)	0.0408 (7)	
H2A	0.4639	0.5334	0.3528	0.049*	
H2B	0.3758	0.4521	0.3885	0.049*	
C3	0.2202 (3)	0.4882 (3)	0.33670 (9)	0.0416 (7)	
H3	0.2097	0.5535	0.3150	0.050*	
C4	0.0533 (3)	0.4714 (3)	0.35987 (9)	0.0439 (7)	
H4A	0.0613	0.4012	0.3791	0.053*	
H4B	−0.0342	0.4545	0.3385	0.053*	
C5	0.0019 (3)	0.5833 (3)	0.38662 (10)	0.0415 (7)	
H5	−0.0158	0.6502	0.3659	0.050*	
C6	−0.1667 (4)	0.5618 (3)	0.40940 (9)	0.0508 (8)	
H6A	−0.2474	0.5302	0.3884	0.061*	
H6B	−0.2097	0.6394	0.4199	0.061*	
C7	−0.1550 (3)	0.4733 (3)	0.44751 (9)	0.0462 (7)	
H7	−0.1227	0.3926	0.4365	0.055*	
C8	−0.0229 (3)	0.5153 (3)	0.48090 (9)	0.0351 (6)	
H8	−0.0600	0.5940	0.4927	0.042*	
C9	0.1503 (3)	0.5350 (2)	0.45808 (8)	0.0342 (6)	
H9	0.1819	0.4558	0.4457	0.041*	
C10	0.1415 (3)	0.6260 (2)	0.41883 (9)	0.0361 (6)	
C11	0.2904 (4)	0.5677 (3)	0.49034 (9)	0.0459 (8)	
H11A	0.2699	0.6494	0.5014	0.055*	
H11B	0.3979	0.5688	0.4750	0.055*	
C12	0.3042 (3)	0.4796 (3)	0.52914 (9)	0.0442 (7)	
H12A	0.3393	0.3998	0.5188	0.053*	
H12B	0.3897	0.5091	0.5492	0.053*	
C13	0.1340 (3)	0.4681 (2)	0.55303 (9)	0.0359 (7)	
C14	−0.0003 (3)	0.4264 (3)	0.51936 (9)	0.0380 (7)	
H14	0.0433	0.3504	0.5067	0.046*	
C15	−0.1512 (4)	0.3896 (3)	0.54761 (10)	0.0517 (8)	
H15A	−0.2190	0.3277	0.5332	0.062*	
H15B	−0.2223	0.4597	0.5539	0.062*	
C16	−0.0713 (4)	0.3389 (3)	0.58960 (10)	0.0576 (9)	
H16A	−0.0917	0.2517	0.5918	0.069*	
H16B	−0.1206	0.3784	0.6149	0.069*	
C17	0.1219 (4)	0.3648 (3)	0.58750 (9)	0.0426 (7)	
H17	0.1754	0.2924	0.5748	0.051*	
C18	0.1007 (4)	0.7560 (3)	0.43452 (10)	0.0541 (8)	
H18A	0.1943	0.7871	0.4511	0.081*	
H18B	0.0010	0.7543	0.4525	0.081*	
H18C	0.0812	0.8078	0.4098	0.081*	
C19	0.0859 (4)	0.5906 (2)	0.57389 (9)	0.0462 (8)	
H19A	0.0679	0.6503	0.5515	0.069*	
H19B	0.1758	0.6172	0.5927	0.069*	
H19C	−0.0162	0.5809	0.5906	0.069*	
C20	0.2012 (4)	0.3833 (3)	0.63295 (9)	0.0481 (8)	
H20	0.1423	0.4517	0.6469	0.058*	
C21	0.1710 (4)	0.2695 (3)	0.66091 (9)	0.0549 (8)	
H21A	0.0532	0.2455	0.6580	0.066*	
H21B	0.2399	0.2034	0.6495	0.066*	
C22	0.2112 (5)	0.2856 (3)	0.70923 (10)	0.0659 (10)	
H22A	0.3244	0.3192	0.7121	0.079*	
H22B	0.1325	0.3441	0.7216	0.079*	
C23	0.2014 (4)	0.1701 (3)	0.73474 (10)	0.0535 (8)	
C24	0.2399 (5)	0.0802 (3)	0.80385 (11)	0.0825 (12)	
H24A	0.1299	0.0429	0.8027	0.124*	
H24B	0.2653	0.1028	0.8334	0.124*	
H24C	0.3236	0.0234	0.7936	0.124*	
C25	0.3888 (4)	0.4142 (4)	0.63165 (12)	0.0798 (12)	
H25A	0.4474	0.3547	0.6143	0.120*	
H25B	0.4336	0.4135	0.6607	0.120*	
H25C	0.4038	0.4938	0.6191	0.120*	
C26	0.1968 (4)	0.3558 (3)	0.27627 (10)	0.0494 (8)	
C27	0.1876 (5)	0.2065 (3)	0.22191 (9)	0.0642 (10)	
H27A	0.0731	0.2362	0.2174	0.077*	
H27B	0.1854	0.1181	0.2201	0.077*	

### Atomic displacement parameters (Å^2^)

**Table:**

	*U*^11^	*U*^22^	*U*^33^	*U*^12^	*U*^13^	*U*^23^
Cl1	0.1037 (8)	0.1095 (8)	0.0566 (5)	−0.0270 (6)	0.0159 (6)	−0.0190 (5)
O1	0.0637 (13)	0.0502 (12)	0.0358 (11)	0.0123 (10)	−0.0067 (11)	−0.0087 (10)
O2	0.0304 (11)	0.126 (2)	0.0641 (15)	−0.0057 (12)	0.0029 (12)	0.0148 (14)
O3	0.0891 (18)	0.0694 (15)	0.0566 (14)	0.0211 (14)	−0.0233 (15)	−0.0142 (12)
O4	0.1050 (19)	0.0487 (12)	0.0433 (12)	0.0106 (13)	−0.0066 (13)	−0.0096 (10)
O5	0.124 (2)	0.0533 (14)	0.0699 (16)	−0.0114 (15)	−0.0356 (17)	0.0132 (13)
O6	0.0814 (17)	0.0611 (13)	0.0445 (12)	0.0078 (12)	0.0031 (13)	0.0078 (11)
C1	0.0495 (19)	0.0389 (16)	0.0421 (16)	−0.0033 (14)	−0.0036 (15)	−0.0019 (14)
C2	0.0362 (15)	0.0453 (16)	0.0410 (16)	−0.0001 (14)	0.0027 (14)	−0.0016 (14)
C3	0.0423 (16)	0.0417 (16)	0.0407 (16)	0.0044 (14)	−0.0009 (15)	−0.0064 (13)
C4	0.0414 (17)	0.0485 (17)	0.0417 (18)	−0.0028 (14)	−0.0091 (15)	−0.0070 (15)
C5	0.0396 (17)	0.0435 (17)	0.0414 (17)	0.0053 (14)	−0.0066 (15)	0.0038 (15)
C6	0.0355 (16)	0.066 (2)	0.0505 (18)	0.0099 (15)	−0.0055 (16)	−0.0017 (16)
C7	0.0289 (15)	0.063 (2)	0.0468 (18)	0.0007 (15)	−0.0016 (15)	−0.0029 (16)
C8	0.0299 (14)	0.0360 (15)	0.0395 (16)	0.0043 (12)	−0.0013 (13)	−0.0072 (13)
C9	0.0304 (14)	0.0336 (15)	0.0387 (15)	0.0060 (12)	−0.0013 (13)	−0.0043 (12)
C10	0.0365 (15)	0.0327 (15)	0.0392 (15)	0.0028 (13)	0.0012 (14)	−0.0045 (13)
C11	0.0340 (16)	0.0604 (19)	0.0434 (17)	−0.0018 (15)	−0.0007 (15)	−0.0012 (15)
C12	0.0372 (16)	0.0516 (17)	0.0438 (17)	0.0012 (14)	−0.0047 (15)	−0.0025 (14)
C13	0.0360 (15)	0.0339 (15)	0.0379 (16)	0.0008 (13)	−0.0009 (14)	−0.0016 (13)
C14	0.0327 (16)	0.0367 (15)	0.0444 (17)	0.0038 (13)	−0.0021 (15)	−0.0075 (14)
C15	0.0417 (17)	0.058 (2)	0.0554 (19)	−0.0081 (15)	−0.0026 (17)	0.0066 (16)
C16	0.0531 (19)	0.063 (2)	0.057 (2)	−0.0165 (16)	−0.0101 (18)	0.0185 (18)
C17	0.0471 (17)	0.0352 (16)	0.0455 (17)	0.0026 (13)	−0.0017 (16)	−0.0015 (14)
C18	0.065 (2)	0.0400 (17)	0.0569 (19)	0.0126 (16)	−0.0005 (18)	−0.0009 (15)
C19	0.0553 (19)	0.0382 (17)	0.0452 (18)	0.0000 (15)	0.0015 (16)	−0.0002 (14)
C20	0.0547 (19)	0.0418 (16)	0.0478 (18)	−0.0083 (15)	−0.0081 (17)	0.0067 (14)
C21	0.059 (2)	0.0509 (19)	0.0547 (19)	−0.0017 (16)	−0.0068 (18)	0.0086 (15)
C22	0.096 (3)	0.050 (2)	0.0516 (19)	−0.0089 (19)	−0.014 (2)	0.0100 (16)
C23	0.055 (2)	0.051 (2)	0.054 (2)	0.0046 (17)	−0.0109 (18)	0.0041 (17)
C24	0.121 (3)	0.072 (2)	0.054 (2)	0.017 (2)	0.002 (2)	0.0246 (19)
C25	0.069 (2)	0.105 (3)	0.065 (2)	−0.031 (2)	−0.023 (2)	0.026 (2)
C26	0.058 (2)	0.049 (2)	0.0415 (18)	0.0017 (17)	0.0030 (18)	−0.0060 (16)
C27	0.091 (3)	0.060 (2)	0.0418 (18)	−0.0061 (19)	0.000 (2)	−0.0203 (16)

### Geometric parameters (Å, º)

**Table:**

Cl1—C27	1.761 (3)	C11—H11B	0.9700
O1—C26	1.327 (3)	C12—C13	1.535 (4)
O1—C3	1.458 (3)	C12—H12A	0.9700
O2—C7	1.433 (3)	C12—H12B	0.9700
O2—H2	0.8200	C13—C19	1.534 (4)
O3—C26	1.185 (4)	C13—C14	1.549 (4)
O4—C26	1.357 (3)	C13—C17	1.551 (4)
O4—C27	1.414 (3)	C14—C15	1.526 (4)
O5—C23	1.191 (4)	C14—H14	0.9800
O6—C23	1.340 (4)	C15—C16	1.538 (4)
O6—C24	1.442 (3)	C15—H15A	0.9700
C1—C2	1.520 (4)	C15—H15B	0.9700
C1—C10	1.537 (4)	C16—C17	1.552 (4)
C1—H1A	0.9700	C16—H16A	0.9700
C1—H1B	0.9700	C16—H16B	0.9700
C2—C3	1.502 (3)	C17—C20	1.542 (4)
C2—H2A	0.9700	C17—H17	0.9800
C2—H2B	0.9700	C18—H18A	0.9600
C3—C4	1.508 (4)	C18—H18B	0.9600
C3—H3	0.9800	C18—H18C	0.9600
C4—C5	1.530 (4)	C19—H19A	0.9600
C4—H4A	0.9700	C19—H19B	0.9600
C4—H4B	0.9700	C19—H19C	0.9600
C5—C6	1.521 (4)	C20—C25	1.519 (4)
C5—C10	1.552 (4)	C20—C21	1.531 (4)
C5—H5	0.9800	C20—H20	0.9800
C6—C7	1.522 (4)	C21—C22	1.526 (4)
C6—H6A	0.9700	C21—H21A	0.9700
C6—H6B	0.9700	C21—H21B	0.9700
C7—C8	1.533 (4)	C22—C23	1.489 (4)
C7—H7	0.9800	C22—H22A	0.9700
C8—C14	1.540 (4)	C22—H22B	0.9700
C8—C9	1.550 (4)	C24—H24A	0.9600
C8—H8	0.9800	C24—H24B	0.9600
C9—C11	1.526 (4)	C24—H24C	0.9600
C9—C10	1.564 (4)	C25—H25A	0.9600
C9—H9	0.9800	C25—H25B	0.9600
C10—C18	1.537 (4)	C25—H25C	0.9600
C11—C12	1.536 (4)	C27—H27A	0.9700
C11—H11A	0.9700	C27—H27B	0.9700
			
C26—O1—C3	115.8 (2)	C12—C13—C17	116.1 (2)
C7—O2—H2	109.5	C14—C13—C17	101.4 (2)
C26—O4—C27	114.9 (3)	C15—C14—C8	120.8 (2)
C23—O6—C24	115.8 (3)	C15—C14—C13	103.4 (2)
C2—C1—C10	115.6 (2)	C8—C14—C13	113.8 (2)
C2—C1—H1A	108.4	C15—C14—H14	105.9
C10—C1—H1A	108.4	C8—C14—H14	105.9
C2—C1—H1B	108.4	C13—C14—H14	105.9
C10—C1—H1B	108.4	C14—C15—C16	104.6 (2)
H1A—C1—H1B	107.4	C14—C15—H15A	110.8
C3—C2—C1	109.2 (2)	C16—C15—H15A	110.8
C3—C2—H2A	109.9	C14—C15—H15B	110.8
C1—C2—H2A	109.9	C16—C15—H15B	110.8
C3—C2—H2B	109.9	H15A—C15—H15B	108.9
C1—C2—H2B	109.9	C15—C16—C17	107.5 (2)
H2A—C2—H2B	108.3	C15—C16—H16A	110.2
O1—C3—C2	107.9 (2)	C17—C16—H16A	110.2
O1—C3—C4	109.2 (2)	C15—C16—H16B	110.2
C2—C3—C4	111.0 (2)	C17—C16—H16B	110.2
O1—C3—H3	109.6	H16A—C16—H16B	108.5
C2—C3—H3	109.6	C20—C17—C13	119.8 (2)
C4—C3—H3	109.6	C20—C17—C16	112.6 (2)
C3—C4—C5	112.7 (2)	C13—C17—C16	102.8 (2)
C3—C4—H4A	109.0	C20—C17—H17	107.0
C5—C4—H4A	109.0	C13—C17—H17	107.0
C3—C4—H4B	109.0	C16—C17—H17	107.0
C5—C4—H4B	109.0	C10—C18—H18A	109.5
H4A—C4—H4B	107.8	C10—C18—H18B	109.5
C6—C5—C4	110.8 (2)	H18A—C18—H18B	109.5
C6—C5—C10	112.0 (2)	C10—C18—H18C	109.5
C4—C5—C10	113.3 (2)	H18A—C18—H18C	109.5
C6—C5—H5	106.8	H18B—C18—H18C	109.5
C4—C5—H5	106.8	C13—C19—H19A	109.5
C10—C5—H5	106.8	C13—C19—H19B	109.5
C5—C6—C7	113.5 (2)	H19A—C19—H19B	109.5
C5—C6—H6A	108.9	C13—C19—H19C	109.5
C7—C6—H6A	108.9	H19A—C19—H19C	109.5
C5—C6—H6B	108.9	H19B—C19—H19C	109.5
C7—C6—H6B	108.9	C25—C20—C21	110.3 (3)
H6A—C6—H6B	107.7	C25—C20—C17	113.6 (3)
O2—C7—C6	105.9 (2)	C21—C20—C17	109.7 (2)
O2—C7—C8	112.7 (2)	C25—C20—H20	107.7
C6—C7—C8	111.3 (2)	C21—C20—H20	107.7
O2—C7—H7	108.9	C17—C20—H20	107.7
C6—C7—H7	108.9	C22—C21—C20	114.7 (2)
C8—C7—H7	108.9	C22—C21—H21A	108.6
C7—C8—C14	113.6 (2)	C20—C21—H21A	108.6
C7—C8—C9	109.8 (2)	C22—C21—H21B	108.6
C14—C8—C9	109.4 (2)	C20—C21—H21B	108.6
C7—C8—H8	107.9	H21A—C21—H21B	107.6
C14—C8—H8	107.9	C23—C22—C21	113.7 (3)
C9—C8—H8	107.9	C23—C22—H22A	108.8
C11—C9—C8	112.2 (2)	C21—C22—H22A	108.8
C11—C9—C10	112.5 (2)	C23—C22—H22B	108.8
C8—C9—C10	113.4 (2)	C21—C22—H22B	108.8
C11—C9—H9	106.0	H22A—C22—H22B	107.7
C8—C9—H9	106.0	O5—C23—O6	122.6 (3)
C10—C9—H9	106.0	O5—C23—C22	125.6 (3)
C18—C10—C1	106.7 (2)	O6—C23—C22	111.7 (3)
C18—C10—C5	109.3 (2)	O6—C24—H24A	109.5
C1—C10—C5	107.8 (2)	O6—C24—H24B	109.5
C18—C10—C9	111.0 (2)	H24A—C24—H24B	109.5
C1—C10—C9	112.7 (2)	O6—C24—H24C	109.5
C5—C10—C9	109.3 (2)	H24A—C24—H24C	109.5
C9—C11—C12	114.0 (2)	H24B—C24—H24C	109.5
C9—C11—H11A	108.7	C20—C25—H25A	109.5
C12—C11—H11A	108.7	C20—C25—H25B	109.5
C9—C11—H11B	108.7	H25A—C25—H25B	109.5
C12—C11—H11B	108.7	C20—C25—H25C	109.5
H11A—C11—H11B	107.6	H25A—C25—H25C	109.5
C13—C12—C11	111.1 (2)	H25B—C25—H25C	109.5
C13—C12—H12A	109.4	O3—C26—O1	128.4 (3)
C11—C12—H12A	109.4	O3—C26—O4	125.3 (3)
C13—C12—H12B	109.4	O1—C26—O4	106.3 (3)
C11—C12—H12B	109.4	O4—C27—Cl1	110.7 (2)
H12A—C12—H12B	108.0	O4—C27—H27A	109.5
C19—C13—C12	110.1 (2)	Cl1—C27—H27A	109.5
C19—C13—C14	111.5 (2)	O4—C27—H27B	109.5
C12—C13—C14	107.7 (2)	Cl1—C27—H27B	109.5
C19—C13—C17	109.7 (2)	H27A—C27—H27B	108.1
			
C10—C1—C2—C3	57.9 (3)	C11—C12—C13—C14	56.6 (3)
C26—O1—C3—C2	−156.1 (2)	C11—C12—C13—C17	169.4 (2)
C26—O1—C3—C4	83.2 (3)	C7—C8—C14—C15	−56.4 (3)
C1—C2—C3—O1	−176.6 (2)	C9—C8—C14—C15	−179.6 (2)
C1—C2—C3—C4	−57.1 (3)	C7—C8—C14—C13	179.6 (2)
O1—C3—C4—C5	175.3 (2)	C9—C8—C14—C13	56.4 (3)
C2—C3—C4—C5	56.5 (3)	C19—C13—C14—C15	−71.7 (3)
C3—C4—C5—C6	−179.9 (2)	C12—C13—C14—C15	167.4 (2)
C3—C4—C5—C10	−53.1 (3)	C17—C13—C14—C15	45.0 (3)
C4—C5—C6—C7	72.7 (3)	C19—C13—C14—C8	61.2 (3)
C10—C5—C6—C7	−54.9 (3)	C12—C13—C14—C8	−59.7 (3)
C5—C6—C7—O2	178.7 (2)	C17—C13—C14—C8	177.9 (2)
C5—C6—C7—C8	55.9 (3)	C8—C14—C15—C16	−161.5 (3)
O2—C7—C8—C14	63.5 (3)	C13—C14—C15—C16	−32.8 (3)
C6—C7—C8—C14	−177.7 (2)	C14—C15—C16—C17	8.1 (3)
O2—C7—C8—C9	−173.5 (2)	C19—C13—C17—C20	−46.8 (3)
C6—C7—C8—C9	−54.7 (3)	C12—C13—C17—C20	78.8 (3)
C7—C8—C9—C11	−175.7 (2)	C14—C13—C17—C20	−164.8 (2)
C14—C8—C9—C11	−50.3 (3)	C19—C13—C17—C16	79.0 (3)
C7—C8—C9—C10	55.5 (3)	C12—C13—C17—C16	−155.4 (2)
C14—C8—C9—C10	−179.1 (2)	C14—C13—C17—C16	−39.0 (3)
C2—C1—C10—C18	−170.0 (2)	C15—C16—C17—C20	149.8 (3)
C2—C1—C10—C5	−52.7 (3)	C15—C16—C17—C13	19.5 (3)
C2—C1—C10—C9	67.9 (3)	C13—C17—C20—C25	−56.5 (4)
C6—C5—C10—C18	−69.4 (3)	C16—C17—C20—C25	−177.5 (3)
C4—C5—C10—C18	164.4 (2)	C13—C17—C20—C21	179.5 (3)
C6—C5—C10—C1	175.0 (2)	C16—C17—C20—C21	58.5 (3)
C4—C5—C10—C1	48.8 (3)	C25—C20—C21—C22	65.9 (4)
C6—C5—C10—C9	52.2 (3)	C17—C20—C21—C22	−168.2 (3)
C4—C5—C10—C9	−74.0 (3)	C20—C21—C22—C23	−172.9 (3)
C11—C9—C10—C18	−62.0 (3)	C24—O6—C23—O5	1.0 (5)
C8—C9—C10—C18	66.7 (3)	C24—O6—C23—C22	−178.4 (3)
C11—C9—C10—C1	57.7 (3)	C21—C22—C23—O5	−0.9 (5)
C8—C9—C10—C1	−173.7 (2)	C21—C22—C23—O6	178.5 (3)
C11—C9—C10—C5	177.5 (2)	C3—O1—C26—O3	5.0 (5)
C8—C9—C10—C5	−53.9 (3)	C3—O1—C26—O4	−175.2 (2)
C8—C9—C11—C12	51.3 (3)	C27—O4—C26—O3	1.9 (5)
C10—C9—C11—C12	−179.4 (2)	C27—O4—C26—O1	−177.9 (2)
C9—C11—C12—C13	−55.0 (3)	C26—O4—C27—Cl1	82.3 (3)
C11—C12—C13—C19	−65.2 (3)		

### Hydrogen-bond geometry (Å, º)

**Table:**

*D*—H···*A*	*D*—H	H···*A*	*D*···*A*	*D*—H···*A*
C11—H11*B*···O2^i^	0.97	2.49	3.339 (4)	146
C27—H27*B*···O5^ii^	0.97	2.40	3.270 (4)	149

Symmetry codes: (i) *x*+1, *y*, *z*; (ii) −*x*+1/2, −*y*, *z*−1/2.
